# Conditional Deletion of Gremlin-1 in Cathepsin K-expressing Mature Osteoclasts Altered the Skeletal Response to Calcium Depletion in Sex-Dependent Manner

**DOI:** 10.1007/s00223-024-01337-7

**Published:** 2025-01-09

**Authors:** Matilda H.-C. Sheng, Charles H. Rundle, David J. Baylink, Kin-Hing William Lau

**Affiliations:** 1 Musculoskeletal Disease Center (151), Jerry L. Pettis Memorial VA Medical Center, VA Loma Linda Healthcare System, 11201 Benton Street, Loma Linda, CA 92357 USA; 2 Department of Medicine and Biochemistry, Loma Linda University School of Medicine, Loma Linda, CA USA

**Keywords:** Gremlin, Osteoclasts, Bone resorption, Calcium deficiency, Regeneration, Gender specific, Bone

## Abstract

This study assessed the novel concept that osteoclast-derived *Grem1* has regulatory functions in the skeletal response to calcium stress using an osteoclastic *Grem1* conditional knockout (cKO) mouse model. The calcium stress was initiated by feeding cKO mutants and wildtype (WT) littermates a calcium-deficient diet for 2 weeks. Deletion of *Grem1* in mature osteoclasts did not affect developmental bone growth nor basal bone turnover. In response to calcium depletion, male cKO mutants showed greater increases in osteoclastic resorption and trabecular bone loss than male WT littermates, indicating an enhanced skeletal sensitivity to calcium depletion in male mutants. The enhanced sensitivity to calcium depletion was sex-dependent, as female cKO mutants showed lower increases in osteoclastic resorption and bone loss than female WT littermates as well as male cKO mutants. The sex disparity in osteoclastic resorption response to calcium stress was intrinsic to osteoclasts since osteoclasts of male but not female cKO mutants showed greater in vitro bone resorption activity than osteoclasts of WT littermates of respective sex. Male cKO mutants displayed smaller bone formation response to calcium depletion than male WT littermates, while female mutants showed bigger bone formation response than female WT littermates, indicating that cKO mutants also displayed sex disparity in bone formation response. The sex disparity in bone formation response was not caused by intrinsic differences in osteoblasts but might be due to sex-dependent differential osteoclastic release of osteogenic factors. In summary, osteoclast-derived gremlin-1 has complicated and sex-dependent regulatory roles in skeletal response to calcium stress.

**Supplementary Information:**

The online version contains supplementary material available at 10.1007/s00223-024-01337-7.

## Introduction

The skeleton plays a central role in calcium homeostasis, as it is the major reservoir for body calcium. Accordingly, calcium stress due to insufficient dietary calcium intake or poor intestinal calcium absorption causes a rapid release of bone calcium to maintain plasma calcium through an increased bone resorption and trabecular bone loss [1–3]. This adaptive homeostatic process, which is referred to as “bone depletion” [4], is achieved through appropriate increments in serum calcitropic hormones (PTH and 1,25(OH)_2_D_3_) in response to the calcium stress [4]. Once the calcium stress is reversed through switching back to calcium-abundant diet, the level of PTH and 1,25(OH)_2_D_3_ returns to normal, and bone formation is quickly increased to eventually restore the lost bone mass and bone strength. This regenerative process is termed “bone repletion” [5]. This tandem mechanism of bone depletion and repletion is an important physiologic regenerative response and is the guardian of adequate bone mass and strength. A deflective bone depletion/repletion mechanism causes significant bone deficit that weakens the skeleton and is a key risk factor for osteoporosis and skeletal fractures [1, 6].

Our recent investigation has made an interesting discovery, in that we found large increases in proliferative cells of osteogenic lineage and an accumulation of multi-layers of osteoblastic cells at marrow sites near the previously resorbed bone surface during the depletion phase [7]. While these osteogenic cells increased the production of collagen matrices, the mineralization of the newly formed matrices was blocked due to insufficient calcium, resulting an increase in osteoid formation and inhibition of bone formation [3, 7]. Upon replenishment of dietary calcium, the multi-layers of osteoblastic cells rapidly migrated to bone surface, where they were activated to promote matrix mineralization and bone formation [7]. Thus, all cellular elements responsible for the subsequent increase in bone formation occurred during the calcium depletion phase. This study focused on the osteoclastic regulation of bone formation during the calcium depletion phase, because information of the osteoclastic regulation of bone formation during calcium depletion is essential to our understanding of the overall regulation of the local bone regeneration process.

The osteoclastic regulation of local bone regeneration is mediated in large parts through the local osteoclastic release of various soluble osteoanabolic factors [8–10]. Our interest in gremlin-1 was initially triggered by the report in a Cambrex microarray database that has since been removed, which showed that human mature osteoclasts exhibited > 100-fold greater expression of gremlin-1 (*GREM1*) than their progenitors. Gremlin-1 is a member of the *Differentially screening-selected gene aberrative in neuroblastoma (Dan)* family of cysteine knot-secreted proteins. Its transcription is upregulated by bone morphogenetic proteins (BMPs) [11]. Gremlin-1 is a multi-functional, regulatory protein that acts through both BMP-dependent and -independent pathways. Accordingly, gremlin-1 is a potent antagonist of BMP-2, 4, 7 and acts as a decoy binding partner for BMPs to prevent the binding of BMPs to their cognate receptors [12]. The gremlin-1-mediated blockage of BMP signaling suppresses the BMP-mediated osteoblast differentiation and bone formation [11]. Gremlin-1 can also act through BMP-independent pathways to enhance endothelial cell migration and neovascularization [13]: gremlin-1 interacts with Wnt in the intestine to activate its canonical signaling, which plays critical functional roles in preventing gut precursor cells from differentiating into mature gut cells and maintaining the activation and self-renewal intestinal stem cells in the human colon crypt [14].

Conditional deletion (cKO) of *Grem1* in mature osteoblasts in mice caused transient increases in trabecular bone mass and bone formation rate (BFR) due to increases in mineral apposition rate (MAR) and not to increases in bone mineralizing surface (TLS), indicating that the increased bone mass was secondary to an increase in osteoblastic activity and not to osteoblastic expansion [15]. Transgenic overexpression of *Grem1* in osteoblasts decreased bone formation, leading to low bone mass and bone fractures [16]. On the other hand, mice with global deletion of *Grem1* (*Grem1* null mice), which were viable in C57BL/6FVB mixed genetic background [17], showed a very different skeletal phenotype, in that *Grem1* null mice exhibited impaired developmental growth and longitudinal bone growth. They developed low bone mass at 4 weeks of age despite an increased bone formation [18]. The low bone mass phenotype, however, was reversed at 3 months of age with the bone formation rate remained elevated. In addition, the restoration effect was sex-dependent, as it was more conspicuous in females than in males [18]. Contrary to osteoblastic *Grem1* cKO mutants, the enhanced bone formation in *Grem1* null mice was secondary to an increase in mineralizing surface (TLS/B.Pm) [18]. The reason for the contrasting bone phenotype between *Grem1* null and osteoblastic *Grem1* cKO mice is unclear, but it may suggest that gremlin-1 derived from osteoclasts could also have key regulatory roles in bone metabolism and in bone formation.

This study sought to test the foregoing hypothesis by characterizing bone and osteoclast phenotypes of cKO mutant mice with conditional deletion of *Grem1* in cathepsin K (*Ctsk*)-expressing mature osteoclasts after 2 weeks of dietary calcium restriction. We postulated that calcium depletion increases expression and secretion of gremlin-1 in mature osteoclasts, which reduces bone resorption through the inhibition of the BMP-dependent activation of osteoclastic resorption [19]. Gremlin-1 can also act indirectly to suppress the canonical Wnt signaling-mediated osteoblastic proliferation and differentiation [16]. This study shows intriguing but complicated sex-dependent regulatory roles for gremlin-1 in the skeletal response to calcium depletion, in that male *Grem1* cKO mutants showed greater increases in bone resorption and trabecular bone loss after 2 weeks of dietary calcium restriction. Conversely, female *Grem1* cKO mutants displaced lower increases in osteoclastic resorption and bone loss.

## Methods

### Animals

Osteoclastic *Grem1* cKO mutants were generated by breeding *Grem1*^*flox/flox*^ mice (generously provided by Professor Richard M. Harland of the University of California at Berkeley) with *CtsK-Cre* mice (obtained through Dr. Laurie Glimcher of the Harvard Medical School). The breeding protocol is outlined schematically in Supplemental Fig. S1. This protocol yielded 25% homozygous mutants (*CtsK-Cre*^+^/*Grem1*^*flox/flox*^), 25% heterozygotes mutants (*CtsK-Cre*^+^/*Grem1*^*flox/−*^), and 50% phenotypically normal WT littermates (*CtsK-Cre*^−^/*Grem1*^*flox/flox*^ or *CtsK-Cre*^*−*^/*Grem1*^*flox/−*^). All mice had free access to food and water and were kept under a 12-h light/12-h dark cycle. The animal work was performed earlier in the animal facility of Loma Linda University and later in the Veterinary Medical Unit (VMU) of the VA Loma Linda Healthcare System. All animal protocols were approved by the Animal Care and Use Committees of the Loma Linda University and the VA Loma Linda Healthcare System as well as the Animal Care and Use Review Office (ACURO) of US Army Medical Research and Materiel Command of the Department of Defense.

### Genotyping Assay

Tail vertebrate tissues (< 2 mm) were taken from each pup at weaning under anesthesia and was digested overnight using the DNeasy kit (Qiagen, San Diego, CA). The PCR-based genotyping protocol for *Grem1* KO mice consisted of a forward primer of 5′-GCA GAA AGA ATG ATA CCA GCC ATC-3′ and a reverse primer of 5′-AAA CTC AGT CAG GAC CCG TCT CTC-3′. The PCR was carried out with a 10-min hot start at 95 °C, followed by 35 cycles of denaturation at 94 °C for 30 s, extension at 59 °C for 30 s, and renaturation at 72 °C for 1 min. Homozygote mutants showed a single 300-bp band, heterozygote mutants showed two bands of 300-bp and 220-bp, respectively, and WT littermates showed a single 220-bp band. The *Ctsk*-Cre transgenic mice were also genotyped by PCR using the following set of three primers: (i) 5′-TTA TTC CTT CCG CCA GGA TG-3′; (ii) 5′-TTG CTG TTA TAC TGC TTC TG-3′; and (iii) 5′-TAG TTT TTA CTG CCA GAC CG-3′. The PCR mix was hot-started at 94 °C for 1.5 min, followed by 35 cycles of denaturation at 95 °C for 30 s, extension 54 °C for 1 min, and renaturation at 72 °C for 1 min. The reaction was terminated with 2 min at 72 °C. The WT littermates showed a single 135 bp product, whereas the *Ctsk*-Cre transgenic mice showed a product of 300 bp with or without the 135-bp product band.

### Dietary Calcium Depletion

Weanling homozygous *Grem1* cKO mutant mice and wildtype (WT) littermates at 4 weeks old were fed a calcium-deficient diet containing < 0.01% calcium and 0.4% phosphate (TD. 95027, Envigo, Livermore, CA) or a calcium-sufficient control diet containing 1.2% calcium and 0.4% phosphate (TD. 97191, Envigo) for 2 weeks as described previously [7]. Animals were euthanized, femurs were isolated, cleaned, and subjected to µ-CT, histology, and histomorphometry analyses.

### Micro-computed Tomography (µ-CT) Analysis

Three-dimensional bone parameters of femurs were assessed with a Scanco vivaCT40 µ-CT scanner (Scanco Medical, Brüttisellen, Switzerland) [20]. Scans were performed at 55 keV with density thresholds segmented at 220–570 mg/cm^3^ hydroxyapatite for trabecular bone and 570–1000 mg/cm^3^ for cortical bone. Both trabecular and cortical bone parameters were measured at the metaphysis of the distal femurs in a region of 0.8 mm in thickness at 10% of the full length form the distal end. The trabecular masks were defined in a semiautomatic manner, starting from the outer mask of the femur and application of 15 erosion cycles to ensure that no cortex was included in the measurement. Total size of cortical porosity was obtained by subtracting cortical bone area from 1.0.

### Bone Histomorphometry

Static bone resorption parameters [osteoclast number per bone surface (N.OC/B.Pm) and osteoclast surface per bone surface (OC.Pm/B.Pm)] were measured at the secondary spongiosa (i.e., 300 µm from the lowest point of growth plate) of distal femur as described in Sheng et al. [21]. For dynamic bone formation parameters [TLS, MAR, and BFR] measurements, mice received calcein or demeclocycline injections, each at 25 mg/kg (i. p.), at the 7th and 13th days of the dietary calcium depletion phase, respectively. One day after the second label, mice were euthanized, and femurs were collected, fixed for 48 h with 10% formalin, dehydrated with ethanol, and embedded into methyl methacrylate (MMA). Thin (15 µm) sections were cut with a Leica Microtome, mounted onto glass slides, and then covered with a coverslip in 70% glycerol. The lengths of single and double labels and distance between double labels on trabecular bone were measured under an Olympus BX53 fluorescence microscope with OsteoMeasure™ (SciMeasure Analytical Systems, Decatur, GA) [22]. TLS = double label length surface + 0.5 × single label length surface; MAR = the distance between double labels/inter-labeling time; and BFR = TLS × MAR.

### Histochemical Staining of Tartrate-Resistant Acid Phosphatase (TRAP)

Femurs, after fixation in formalin for 2 days, were soaked in graded (70%, 85%, and 95%) Glycol methacrylate (GMA) solution (Polysciences Inc., PA) and embedded into GMA. After polymerization, GMA blocks were kept at cold until sectioning. Continuous thin sections of 3.5 µm in thickness were cut with Leica Microtome. Slides with bone specimens were air dried, baked for 30 min at 60 ℃, and stored at 4 ℃. Upon staining, specimens were incubated with Naphthol AS-BI phosphate substrate solution in sodium acetate buffer (pH 4.8) for 1 h at 37 ℃ followed by color development with 2.5% paraosaniline chloride and 2% sodium nitrite. Specimens were counter-stained with 0.4% toluidine blue for 10 s or methyl green for 30 s.

### Osteoclastic Cell Cultures

After removal of muscle and connective tissues from femurs and tibiae, bone shells were rinsed thoroughly and cut open at both ends. Marrow cells were collected by flushing out with culture medium using a 26-G syringe after which marrow cells were treated with red blood cells (RBCs) lysis solution to remove RBCs. After 48 h, the supernatant of the cultures was collected and filtered through a cell strainer and counted. Resorption pit formation assay was performed as described in Suhr et al. [23], a total of 1 × 10^5^ marrow cells were plated into a 24-well plate containing a bone disk and cultured for 9 days in the presence of 40-ng/mL mCSF (GenScript, Piscataway, NJ) and 50-ng/mL recombinant mouse RANKL (Santa Cruz Biotech, Santa Cruz, CA). For PCR-based gene expression studies, 1.6 × 10^6^ marrow cells were seeded into a 12-well plates with duplicates per genotype and were repeated three times.

### In Vitro Cell Proliferation Assays

For cell proliferation assay, a total of 5,000 cells per well of 96 wells were plated overnight. Cells were cultured in α-MEM containing 1% FBS with or without added conditioned medium (CM) at 1:2 dilution collected from the cultures of osteoclasts. Cells were then exposed to 10 µM of 5-bromo-2′-deoxyuridine (BrdU) during the last 16 h of the 24 h culturing. The BrdU incorporation was determined with a commercial ELISA kit according to protocol provided by the manufacturer (Roche or Sigma/Millipore, St. Louis, MO). In some experiments, the relative cell number (as a surrogate of cell proliferation) was indirectly determined with the 3-(4,5-dimethylthiazol-2-yl)-2,5-diphenyltetrazolium bromide (MTT) activity colorimetric assay (Sigma-Aldrich, St. Louis, MO), which assesses cell metabolic activity as an index of the number of viable cells.

### Alkaline Phosphatase (ALP) Activity Assay

Osteoblasts were plated into a 24-well plate at a density of 10^4^ cells per cm^2^ and cultured in 1% fetal bovine serum (FBS) with or without the osteoclast CM (at 1:2 dilution) for 48 h. Cell layers were rinsed once with phosphate-buffered saline (PBS) and extracted with 0.1% Triton X-100. Protein was determined with the Pierce BCA protein assay kit (Thermo Fisher Scientific, San Diego, CA). ALP activity was determined by the increase in absorbance at 405 nm over time after the incubation of cellular lysis with the substrate solution containing p-nitrophenyl phosphate and MgCl_2_ in carbonate–bicarbonate buffer (pH 10.3). ALP-specific activity was calculated by dividing the change in absorbance per min by µg of total cellular protein.

### Mineralized Nodule Formation Assay

Briefly, a total of 8 × 10^4^ osteoblasts (isolated from calvarias of 6 days-old C57BL/6J mice) per well of a 12-well plate were cultured in α-MEM containing 10-mM β-glycerophosphate, 50-µg/mL ascorbate, 5% FBS, and 50% CM of osteoclasts derived from *Grem1* cKO mutants or WT littermates of both sexes. Culture medium was changed every three days for 21 days. Mineralized nodules were visualized by Von Kossa staining and imaged with a Zeiss microscope. The total area of mineralized nodules covering the entire well was quantified using the ImageJ software.

### Immunohistochemical (IHC) Staining Assays

Paraffin-embedded bone sections were baked for 1 h to enhance the attachment, and paraffin wax was removed through three changes of xylene. After rehydration, bone specimen was blocked with 3% hydrogen peroxide (H_2_O_2_) for 20 min at room temperature and heated in citrate buffer (pH 6.0) at 95 °C. After 30 min of blocking with normal serum, specimens were incubated with rabbit anti-mouse gremlin-1 antibody (Bioss, Woburn, MA) at 4 °C overnight. The HRP-conjugated anti-rabbit antibody (Sigma/Millipore, St. Louis, MO) and DAB/H_2_O_2_ (Abcam) were applied to visualize positive cells. All sections were counter-stained with hematoxylin. The cell count was performed under a bright-field Olympus BX53 microscope using the OsteoMeasure™ system.

### Reverse Transcriptase Quantitative Polymerase Chain Reaction (RT-qPCR) Gene Expression Assays

Briefly, tibiae (including bone marrow) were pulverized using pre-chilled mortars/pestles with liquid nitrogen. Qiazol Lysis Reagent (Ambion, Austin, TX) and chloroform were used to isolate total RNA, which was further purified with an RNeasy Mini kit (Qiagen, Valencia, CA). For cell cultures, cells were lysed in Qiazol Lysis Reagent and extracted with chloroform and further purified with RNeasy Mini kit. cDNA was generated from RNA samples of tibia (or osteoclast cultures) by reverse transcription using GenScript M-MuLV Reverse Transcriptase kit (GenScript, Piscataway, NJ). The relative level of *Grem1* (normalized against β-actin) was determined by the Cycle threshold method using a SyBr Green-based PCR kit (Applied Biosystems, Foster City, CA) or GoTaq qPCR master Mix (Promega) in an ABI 7500 Fast cycler (Applied Biosystems) or a CFX96 Real-Time System (Bio-Rad Labs, Hercules, CA).

### Statistical Analysis

Results were shown as mean ± standard error of the mean (SEM). Statistical significance of the differences among test groups was determined with two-tailed Student’s *t* test or one- or two-way analysis of variance (ANOVA). Paired two-tailed *t* test was used to determine resorption pit size or osteoclast cell size. *P* < 0.05 considers statistically significant.

## Results

### Upregulation of *Grem1* Expression in Mature Osteoclasts

**Fig. 1:**
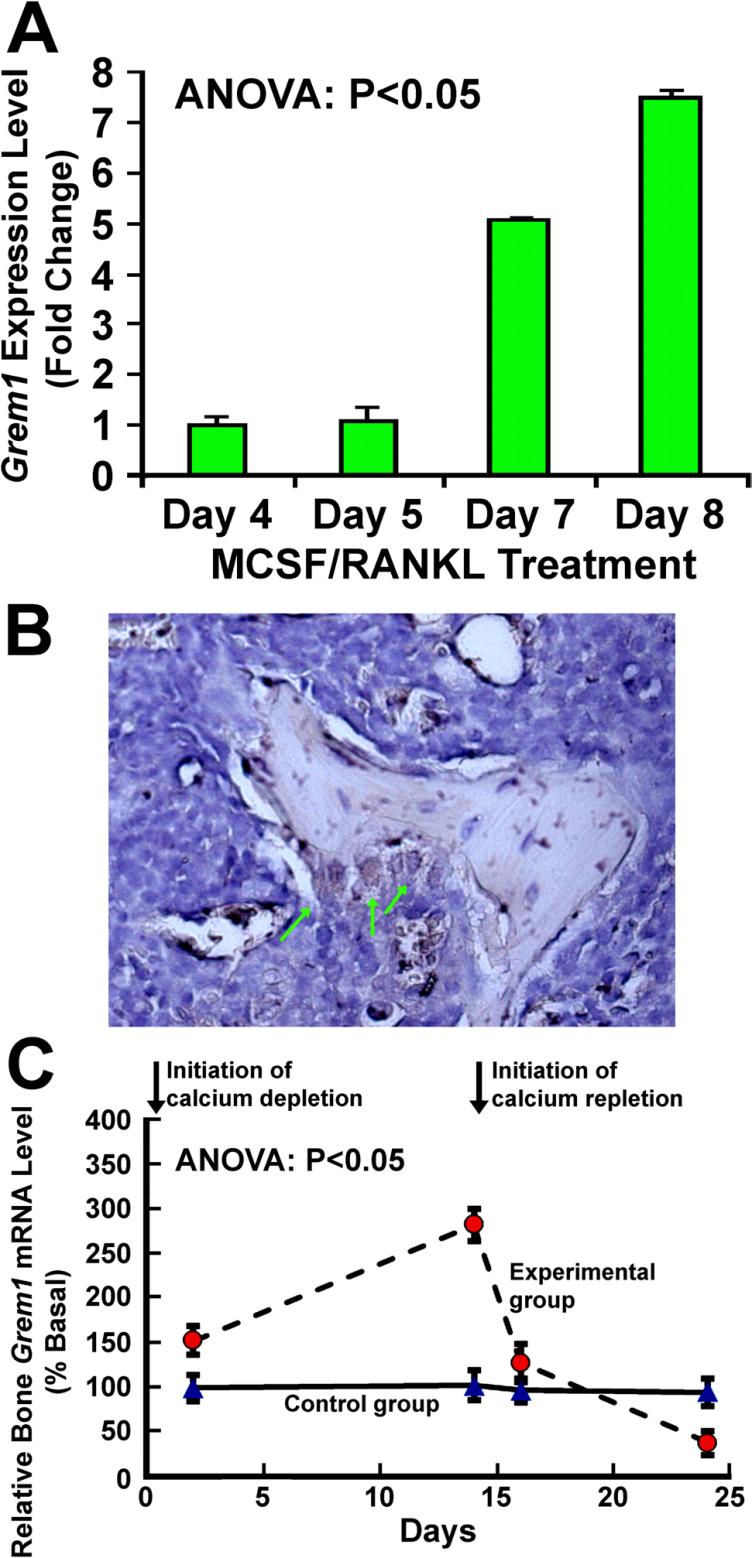
Mature osteoclasts express gremlin-1 and its expression is upregulated by osteoclastic differentiation and calcium depletion. In **A**, the expression of *Grem1* in marrow-derived osteoclasts from groups of three young adult male C57BL/6J mice was determined by RT-qPCR. The expression of *Grem1* was normalized by β-actin. Data are presented as fold change from day 4. Statistical significance was determined with one-way ANOVA. In **B**, a thin section of femur of a representative WT control mouse was stained for expression of gremlin-1 protein using an anti-gremlin-1 antibody by IHC and counter-stained with hematoxylin. Gremlin-1 was stained in brownish color. Green arrows indicate active osteoclasts on resorbing surface within the resorption lacune. Scale bars = 20 µm. In **C**, two groups of 4-weeks old male C57BL/6J mice were fed 14 days of low calcium (< 0.01%) diet followed by 10 days of high calcium (1.2%) diet (Experimental group). The control group of mice were fed high calcium diet throughout to control for age effects. At day 2, 14, 16, and 24 days, the femur of randomly selected two mice per group was isolated and total RNA was prepared. Relative *Grem1* mRNA levels (normalized against *β-actin* mRNA) were determined by RT-qPCR. Results are shown as relative % of the control mice at day 2. Data are shown as mean ± SEM. Statistical significance was determined with one-way ANOVA. Due to the minimal number of replicates in each time point, statistical significance at each individual time point was not determined

The cellular *Grem1* mRNA level in marrow-derived osteoclast precursors at the 4th and 5th day of the eight days mCSF/RANKL treatment was similar but it was upregulated by > fourfold and > sevenfold of that of the 5th day at 7th and 8th days, respectively (Fig. 1A), at which time large numbers of multinucleated (≥ 3 nuclei) osteoclasts were formed. Immunohistochemical staining of femoral thin sections for gremlin-1 confirmed the presence of gremlin-1 protein in osteoclasts along the resorbing bone surface (Fig. 1B), confirming that mature osteoclasts expressed gremlin-1.

Calcium deficiency induces osteoclast differentiation and activation [2, 3]. Thus, we performed a pilot study to determine if calcium deficiency would upregulate *Grem1* expression in bone. We measured the relative *Grem1* mRNA levels in femurs of male weanling C57BL/6J mice after they were on a calcium-deficient diet (< 0.01% calcium) for 14 days followed by a calcium-sufficient diet (1.2% calcium) for 10 additional days. The bone *Grem1* mRNA level was increased 0.5-fold and ~ twofold, respectively, at day 2 and 14 of the calcium depletion phase (Fig. 1C). It rapidly returned to basal level within 2 days of calcium repletion (day 16) and was then decreased to ~ 25% of basal level after 10 days of repletion (day 24).

**Fig. 2:**
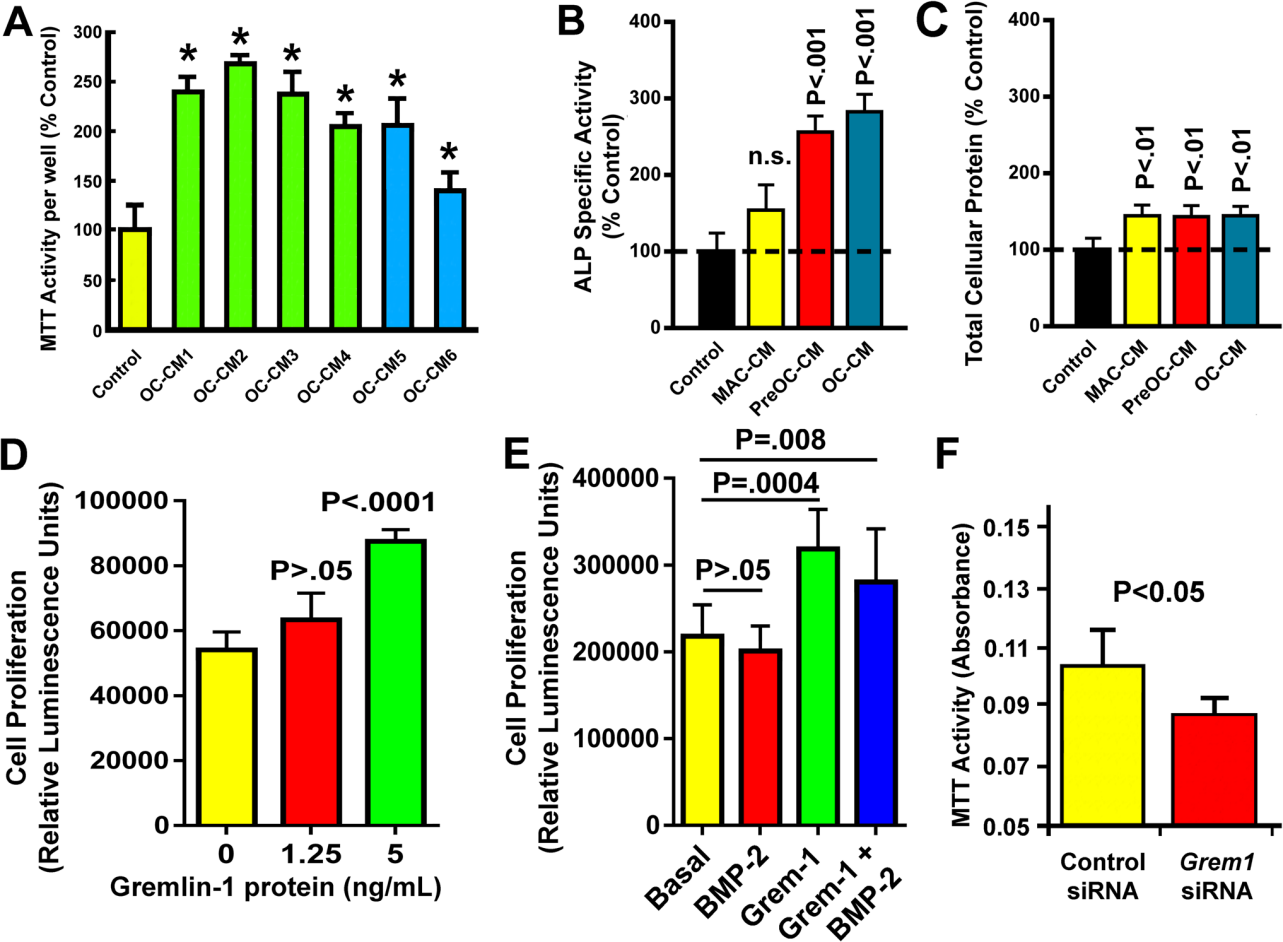
Mouse marrow-derived osteoclasts released into CM soluble osteogenic factors that include gremlin-1 to promote proliferation and osteogenic differentiation of murine MC3T3-E1 osteoblastic cells. In **A**, CM of marrow-derived mouse osteoclasts derived from six young adult C57BL/6J mice [four males (OC-CM1-4) and two females (OC-CM5-6)], at 50% concentration (in the presence of 5% FBS), were added to MC3T3-E1 cells for 48 h. The metabolic activity of viable cells was then determined with the MTT assay (as a surrogate assay for cell proliferation). The control is 5% FBS in α-MEM. Results are shown as the % of the control (five replicates per group and six repeat measurements). *P < 0.001, compared to control. In **B** and **C**, pooled CMs of mouse macrophages (MAC-CM), osteoclast precursors (PreOC-CM), and differentiated osteoclasts (OC-CM), each at 50% concentration, were added to MC3T3-E1 osteoblasts. Osteoclasts (6–7 days in the mCSF/RANKL treatment), pre-osteoclasts (3 days in the mCSF/RANKL treatment), and macrophages (mCSF alone) were isolated from C57BL/6J mice according to established procedures. Cellular ALP-specific activity (**B**) and total cellular protein (**C**) were measured 48 h. after incubation. Results are shown as % of the control (e.g., 5% FBS in α-MEM), mean ± SEM, n = 6 per group. In **D**, the effect of 24-h incubation of MC3T3-E1 cells with recombinant gremlin-1 protein at 1.25 or 5 ng/mL on BrdU incorporation (as an index of cell proliferation; Roche BrdU Kit). BrdU was added during the final 6 h of the incubation. In **E**, MC3T3-E1 cells were treated with 10 ng/mL of BMP-2, 5-ng/mL gremlin-1, or both BMP-2 and gremlin-1 protein for 24 h. BrdU incorporation was determined as in panel (**D**). In **F**, MC3T3-E1 cells were treated with CM (at 50% concentration) of osteoclasts transfected with *Grem1* shRNA or control shRNA for 48 h, and cellular metabolism of each treated cells were measured with the MTT activity. In **D**–**F**, results are shown as mean ± SEM, *n* = 6 per group. Statistical significance was determined with two-tailed Student’s *t* test. *P* > 0.05 = statistically not significant

Active osteoclasts released soluble bone cell mitogens to modulate bone metabolism [10]. Accordingly, we next determined if osteoclasts derived from four male (OC-CM1-4) or two female (OC-CM5-6) C57BL/6J mice released into CM soluble osteogenic factors and if recombinant gremlin-1 has osteogenic activity. Figure 2A shows that each test CM increased MTT activity by 1.5-fold to 2.5-fold in MC3T3-E1 osteoblastic cells. CM of mature osteoclasts (OC-CM) and that of osteoclast precursors (PreOC-CM), but not CM of macrophages (MAC-CM), increased cellular ALP-specific activity in stromal cells after 48 h incubation (Fig. 2B), albeit all three CMs increased cellular protein levels (Fig. 2C). However, it is unclear if the increased ALP-specific activity was due to an earlier onset of differentiation as the result of an increased osteoblastic proliferation or to a direct enhancement of osteoblastic differentiation independent of osteoblast proliferation. Nevertheless, these findings indicate that the osteogenic activity was restricted to OC-CM and PreOC-CM. In addition, recombinant gremlin-1 at 1.25 ng/mL and 5 ng/mL increased MTT activity in MC3T3-E1 cells in an apparent dose-dependent manner (Fig. 2D). However, BMP-2 only slightly but not significantly suppressed BrdU incorporation (Fig. 2E). CM of osteoclasts treated with *Grem1* siRNA (decreased *Grem1* expression by ~ 50%) reduced MTT activity in mouse stromal cells by ~ 35% compared to CM of control siRNA-treated osteoclasts (Fig. 2F), suggesting that osteoclast-derived gremlin-1 might have contributed to the osteogenic activity in CM os osteoclasts.

### Conditional Knockout (cKO) of *Grem1 *in Ctsk-Expressing Cells Enhanced in Male Mutants But Reduced in Female Mutant Mice the Skeletal Sensitivity to Calcium Depletion With Respect to Trabecular Bone Loss

*Grem1* expression levels in bone of 6-week-old male and female osteoclastic *Grem1* cKO mutant mice were both reduced by ~ 70% (Supplemental Fig. S2A). Deletion of *Grem1* in Ctsk-expressing cells also had no effects on body weight (Supplemental Fig. S2B) or femur length (Supplemental Fig. S2C) in both male and female cKO mutants, indicating that deficient in osteoclast-derived gremlin-1 did not affect developmental bone growth.

**Fig. 3:**
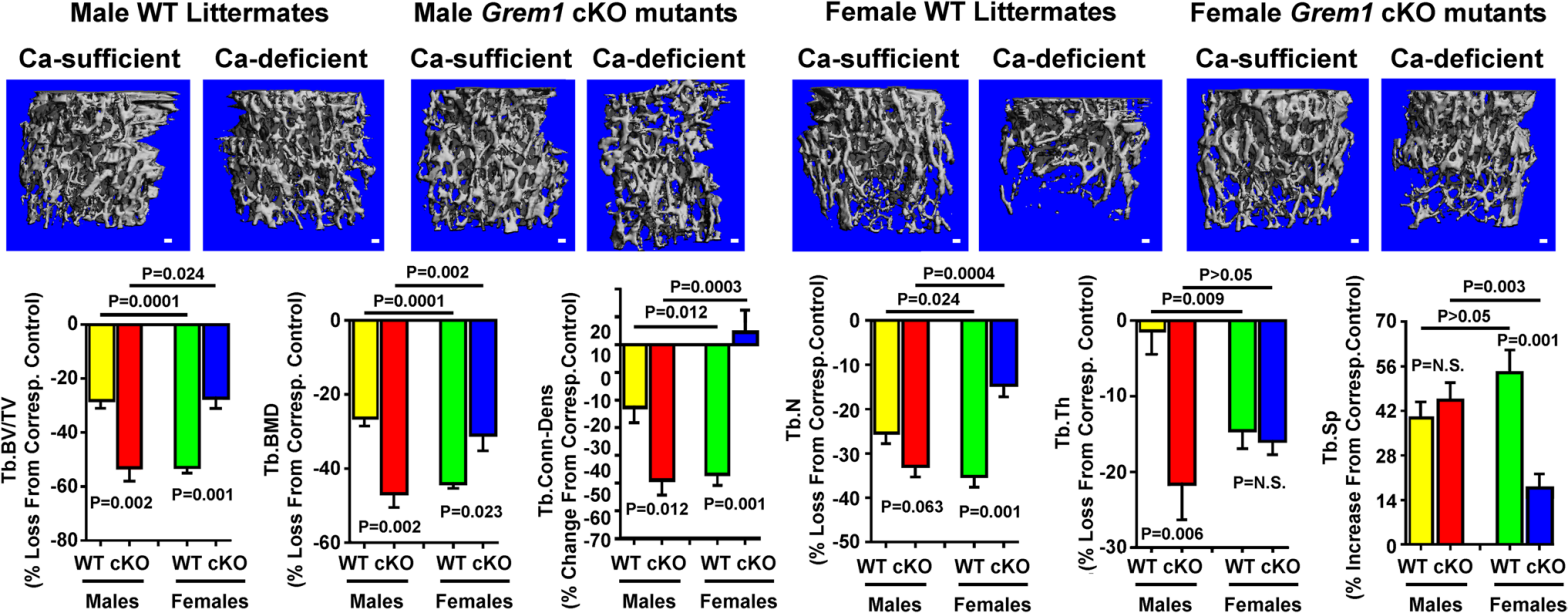
µ-CT evidence that weanling *Grem1* cKO mutant mice and WT littermates exhibited contrasting sex-dependent trabecular bone loss response to 2 weeks of dietary calcium depletion. Male and female *Grem1* cKO mice at weanling (4 weeks of age) as well as weanling WT littermates of respective sex were fed a calcium-deficient diet for 2 weeks. Parallel groups of weanling male and female cKO mice and WT littermates were fed a calcium-sufficient diet for 2 weeks as corresponding controls for comparison. Trabecular bone parameters were determined at the secondary spongiosa of distal femur by µ-CT. Top panel shows a representative photomicrograph of the 3D reconstruction of trabecular bone at the AP view of representative male and female mouse of *Grem1* cKO mutants or corresponding WT littermates. Scale bars = 100 µm. Bottom summarizes the quantitative results. Results are reported as relative % loss from corresponding calcium-sufficient control group (mean ± SEM, *n* = 6–9 per group). Statistical analyses were performed with two-way ANOVA followed with Tukey post hoc test. *P* > 0.05 = statistically not significant

Deletion of *Grem1* in mature osteoclasts did not have significant effects on basal trabecular bone parameters, determined by µ-CT, in either male or female cKO mutants at 6 weeks of age (Supplemental Fig. S3). However, after 2 weeks of dietary calcium depletion, female WT mice showed greater losses of trabecular bone volume/tissue volume (Tb.BV/TV), bone mineral density (Tb.BMD), connectivity density (Tb.Conn-Dens), number (Tb.N), and thickness (Tb.Th), as well as a bigger increase in separation (Tb.Sp) compared to male WT mice (Fig. 3). This observation is consistent with a previous report that normal female rats had greater response to calcium deficiency with respect to trabecular bone loss than normal male rats [24]. Conversely, male cKO mutants showed much bigger relative losses in Tb.BV/TV, Tb.BMD, Tb.Conn-Dens, and Tb.Th than male WT littermates, whereas female cKO mutants lost significantly less trabecular bone mass and density than female WT littermates, indicating that male osteoclastic *Grem1* cKO mutants had an enhanced skeletal response to calcium deficiency, while female cKO mutants not only did not show an enhanced response and might even show substantially reduced sensitivity to calcium deficient-induced trabecular bone loss than both male cKO mutants and female WT littermates (Fig. 3). Accordingly, there was an apparent reverse shift in sex-dependent sensitivity to calcium depletion upon deletion of *Grem1* in mature osteoclasts. Because of this apparent reverse shift in the sex disparity in the skeletal sensitivity to calcium deficiency in cKO mutants, the overall trabecular bone loss to calcium depletion in male *Grem1* cKO mice was greater than that in female cKO mutants.

**Fig. 4:**
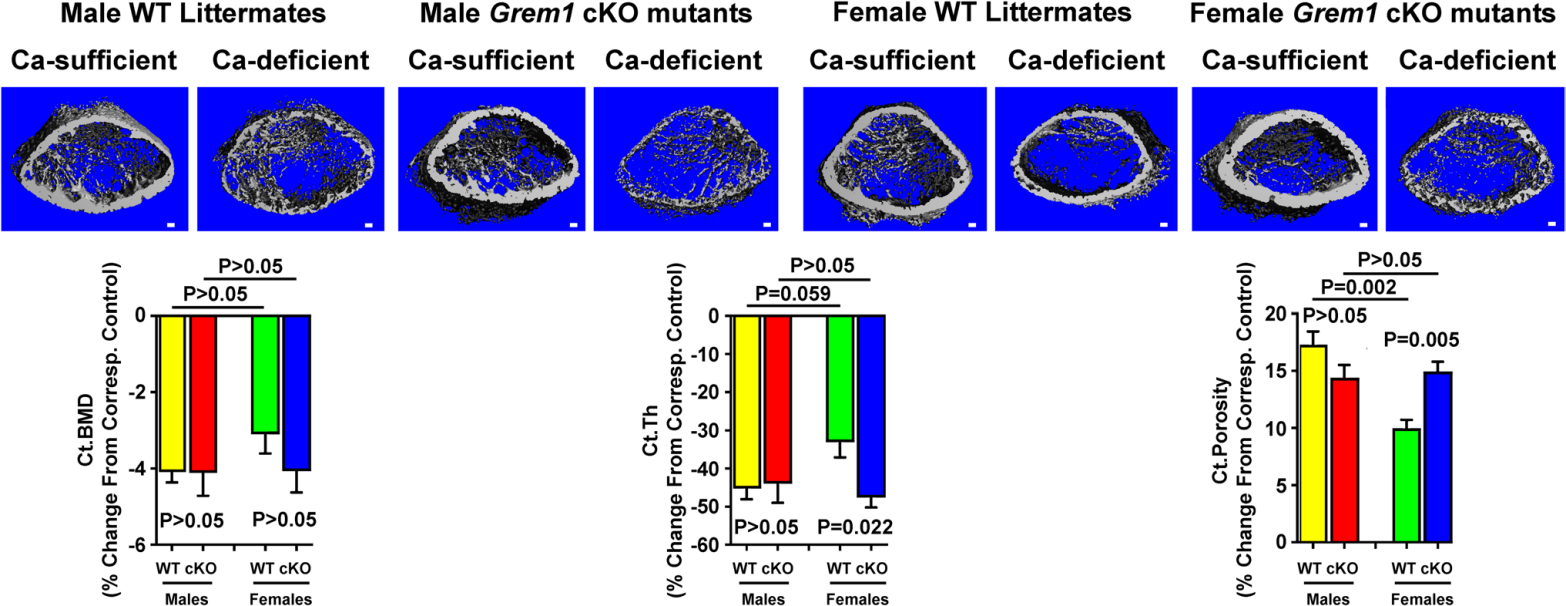
µ-CT evidence that weanling *Grem1* cKO mutant mice and WT littermates exhibited contrasting sex-dependent cortical bone loss response to 2 weeks of dietary calcium depletion. Cortical bone was contoured around the cortical shell for measurements by µ-CT. Top panel shows a representative photomicrograph of the 3D reconstruction of cortical bone shell of representative male and female mouse of *Grem1* cKO mutants or corresponding WT littermates. Scale bars = 100 µm. Bottom summarizes the quantitative results. Results are reported as relative % loss from corresponding calcium-sufficient control group (mean ± SEM, *n* = 6–9 per group). Statistical analyses were performed with two-way ANOVA followed with Tukey post hoc test. *P* > 0.05 = statistically not significant

As previously reported [7], the 2 weeks of dietary calcium restriction also caused substantial losses in cortical bone. Accordingly, the calcium depletion caused similar changes in cortical bone density (Ct.BMD), thickness (Ct.Th), and porosity (Ct.Porosity) in both male WT and male cKO mutants. However, the female cKO mutants displayed a greater loss of Ct.Th and a greater increase of Ct.Porosity in skeletal response to calcium depletion than female WT littermates (Fig. 4). The calcium deficiency-induced cortical bone loss was similar in male and female cKO mutants, indicating a lack of sex disparity in the calcium depletion-induced decrease in cortical bone mass in *Grem1* cKO mutants.

### Conditional Deletion of *Grem1* in Osteoclasts Increased Osteoclastic Resorption and Suppressed Bone Formation After 2 Weeks of Dietary Calcium Depletion in Male But Not Female cKO Mutants

**Fig. 5:**
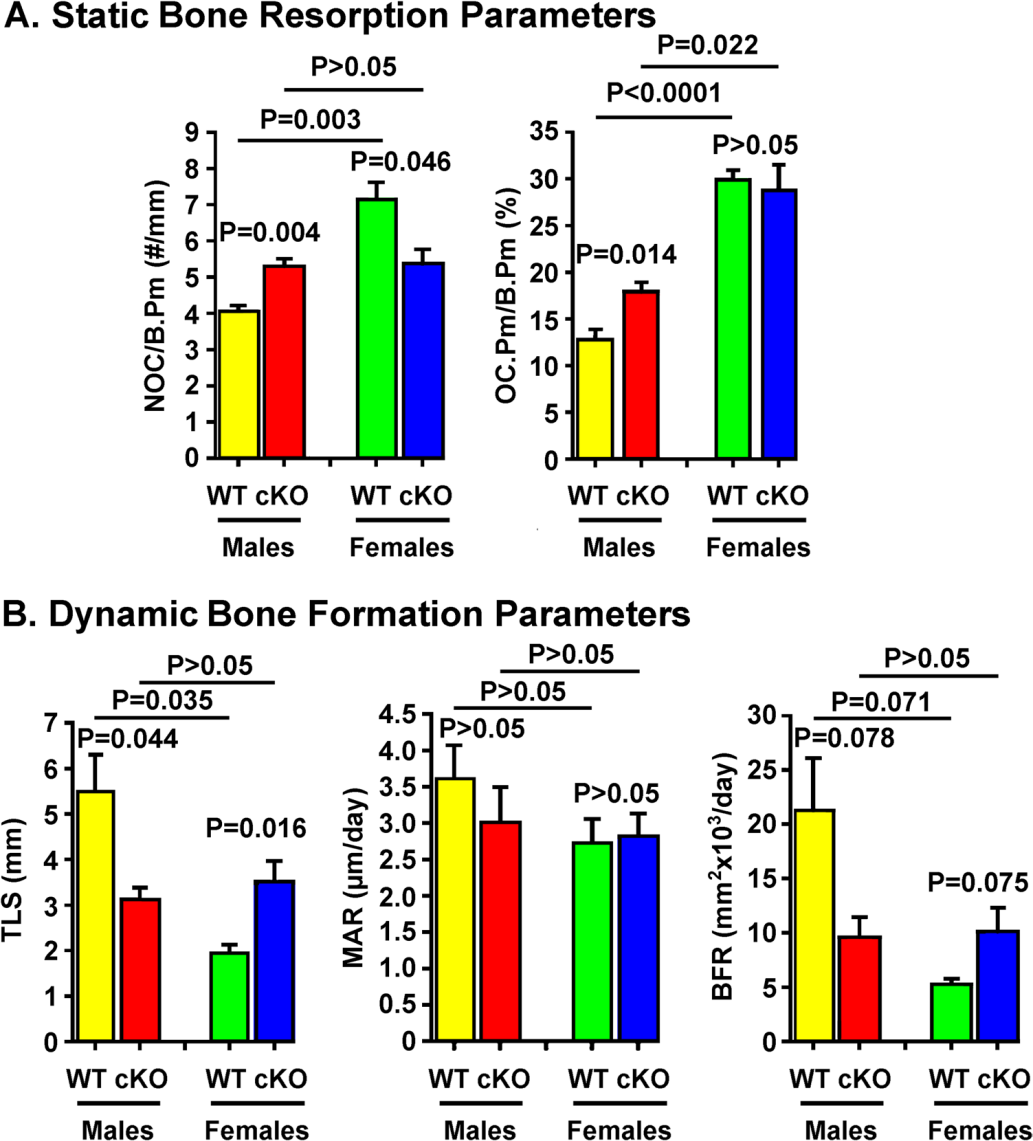
Dietary calcium depletion increased bone resorption (**A**) and suppressed bone formation (**B**) in *Grem1* cKO mutant mice in sex-dependent fashions. In **A**, static bone resorption parameters, i.e., NOC/B.Pm and OC.Pm/B.Pm, were determined by bone histomorphometry at the secondary spongiosa of distal femur of male and female *Grem1* cKO mice and corresponding WT littermates of corresponding sex after 2 weeks of dietary calcium depletion. Results are reported as mean ± SEM (*n* = 5–7 per group). In **B**, at the 7th and 13th days of the two-week dietary calcium depletion phase, each mouse received 25 mg/kg (i.p.) of calcein and demeclocycline, respectively. Dynamic bone formation parameters, i.e., TLS, MAR, and BFR, were determined by bone histomorphometry at secondary spongiosa of distal femur. Results are reported as mean ± SEM (*n* = 5–7 per group). Statistical analyses were performed with two-way ANOVA followed with Tukey post hoc test. *P* > 0.05 = statistically not significant

Bone histomorphometry revealed that conditional deletion of *Grem1* in osteoclasts did not affect static resorption or dynamic bone formation parameters significantly under basal conditions (Supplemental Fig. S4). However, after 2 weeks of calcium depletion, male cKO mutants had greater NOC/B.Pm and OC.PM/B.PM compared to male WT littermates (Fig. 5A). Surprisingly, NOC/B.Pm but not OC.Pm/B.Pm in female cKO mutants was less than that in female WT littermates. These findings indicate that calcium depletion enhanced osteoclast resorption in male mutants but reduced osteoclast resorption in female mutants, confirming a converse sex disparity to calcium depletion-induced osteoclast activation.

With respect to dynamic bone formation parameters, BFR of male WT mice at 6 weeks of age was higher after calcium depletion than that of female WT mice, which was due to greater TLS without significant change on MAR (Fig. 5B). Conversely, calcium depletion in male cKO mutants reduced TLS by 43% (*P* = 0.044) with a small and not significant reduction in MAR, resulting in 55% reduction in BFR (*P* = 0.078) than male WT littermates. However, the same calcium depletion in female cKO mutants not only did not reduce but instead increased BFR by 93% (*P* = 0.075) that was caused primarily by an increased TLS (by 81%, *P* = 0.016). Accordingly, these cKO mutants also displayed sex-specific contrasting responses in dynamic bone formation to calcium depletion. BFR, MAR, and TLS of male cKO mutants were similar to those of female cKO mutants. Thus, it may be interpreted that deficient expression of *Grem1* in mature osteoclasts abolished sex disparity in BFR response to calcium deficiency as that seen in WT littermates.

### Deficient *Grem1* Expression in Osteoclasts Enhanced Activation of Osteoclasts Derived From Both Male and Female cKO Mutants

**Fig. 6:**
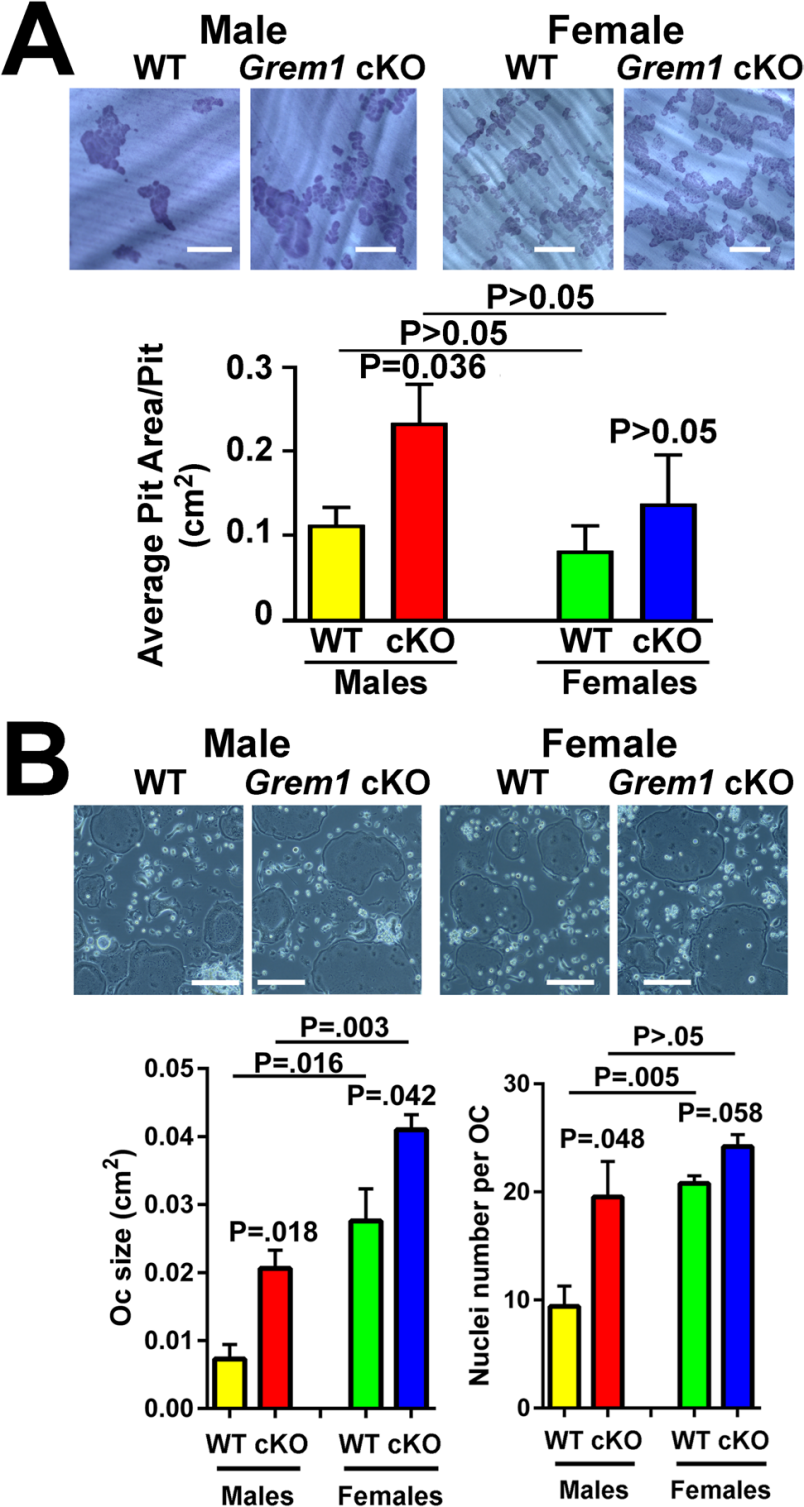
Marrow-derived osteoclasts of male but not female *Grem1* cKO mutants exhibited greater bone resorption activity (**A**), were larger and contained more nuclei (**B**) than osteoclasts of age- and sex-matched WT littermates in vitro. **A** Marrow-derived osteoclast precursors were plated on thin dentine slices and treated with m-CSF and recombinant RANKL for 9 days. Cells on the dentine slices were removed by sonication, and resorption pits were lightly stained with hematoxylin to aid identification. The resorption pit area per pit was measured with the ImageJ software. Top shows micrograph of representative resorptive pits created by osteoclasts of a representative male and female *Grem1* cKO mutant and male and female WT littermate, each. Bottom summarizes the quantitative results. Results are reported as mean ± SEM (*n* = 3–4 per group). Scale bars = 100 µm. Statistical analyses were performed with two-tailed Student’s *t* test. *P* > 0.05 = statistically not significant. **B** Marrow-derived osteoclast precursors at a density of 4 × 10^6^ cells per well of a 24-well culture plate were plated in each well and treated with mCSF/RANKL for 7–9 days. The area of multinucleated osteoclasts was measured with ImageJ software and the number of nuclei was counted with the aid of microscope. Top shows a representative photomicrograph of osteoclasts of each of a male and a female cKO mutant and a male and a female WT littermate. Bottom summarizes the average osteoclast size (left) and average number of nuclei per osteoclast (right). Results are reported as mean ± SEM (*n* = 3–4 per group). Scale bars = 20 µm. Statistical analyses were performed with two-tailed Student’s *t* test. *P* > 0.05 = statistically not significant

To determine if the sex disparity on osteoclastic resorption of *Grem1* cKO mutants was intrinsic to their osteoclasts, the bone resorption activity of marrow-derived osteoclasts of male and female cKO mutants was determined and compared with that of osteoclasts of male and female WT littermates using an in vitro resorption pit formation assay (Fig. 6A). The average resorption pit area per pit (an estimate of average resorption activity per osteoclast) created by male and female *Grem1* cKO mutant osteoclasts was greater by 112% and 62%, respectively, than those created by osteoclasts of WT littermates of corresponding sex. The average cell size and number of nuclei of marrow-derived osteoclasts of male and female *Grem1* cKO and WT littermates were also measured, because the relative size of an osteoclast [25, 26] and the number of nuclei per osteoclast [27] were each correlated positively with the resorption activity of the osteoclast. Accordingly, the average size of osteoclasts of female WT and female cKO mutant mice was each larger than that of osteoclasts of male and male WT littermates, respectively. The average size of osteoclasts of male and female cKO mutants was also greater by 182% and by 49%, respectively, than male and female WT osteoclasts. Similarly, the average number of nuclei per osteoclast in male and female cKO was each more than that in osteoclasts of male and female WT littermates (Fig. 6B).

### Deficient *Grem1* Expression in Osteoclasts Enhanced Bone Formation Through Osteoclastic Release of Soluble Osteogenic Factors

**Fig. 7:**
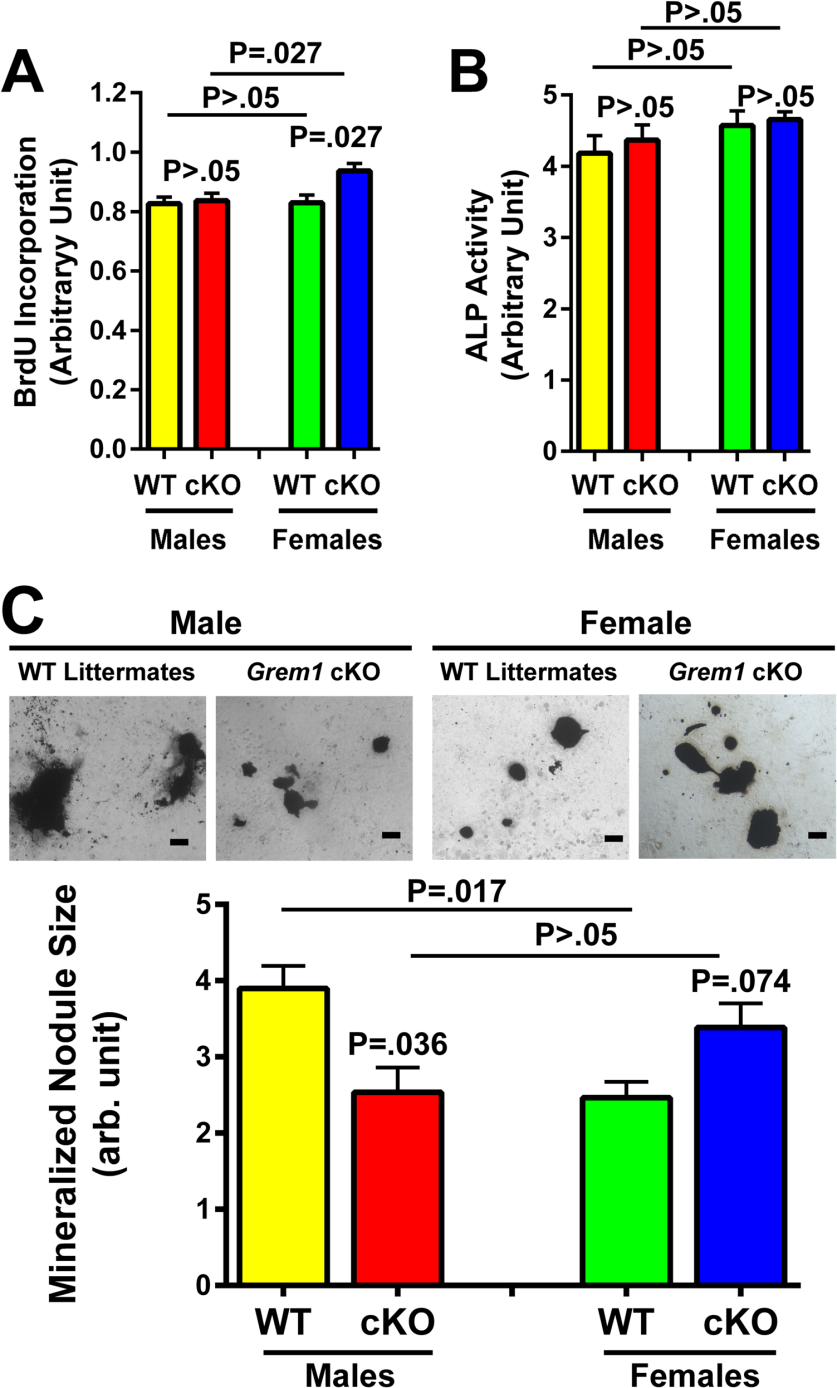
Conditional media (CM) of osteoclasts of male *Grem1* cKO mutants released less soluble osteoanabolic factors than male WT osteoclasts, while that of female cKO mutant osteoclasts released more soluble osteoanabolic factors than female WT osteoclasts. **A** CM of cultures of osteoclasts, derived after 7–9 days of c-MSF/RANKL treatment, of male and female Grem1 cKO mutants or male and female WT littermates. The resulting CM at 1:1 dilution was added to cultures of primary osteoblasts of C57BL/6J mice and were incubated for 24 h for BrdU incorporation (*n* = 6 per group with the Sigma BrdU Assay kit), 48 h for cellular ALP activity (*n* = 4 per group), or 21 days for mineralized nodule formation (*n* = 3 per group). In **A** and **B**, results are reported as mean ± SEM. In **C**, top shows photomicrograph of mineralized nodule formed by a representative male and female *Grem1* cKO mutant or WT littermate each. The average area of mineralized nodules was measured with ImageJ software. Scale bars = 100 µm. Results are reported as mean ± SEM. Statistical analyses were performed with two-tailed Student’s *t* test. *P* > 0.05 = statistically not significant

To evaluate if deficient expression of osteoclast-derived gremlin-1 caused differential release of soluble osteogenic factors to alter bone formation, we compared the ability of CMs (1:1 dilution) of male and females cKO mutants with those of male and female WT littermates on the proliferation (by measuring BrdU incorporation) and osteoblastic differentiation (by assaying cellular ALP activity) of primary mouse osteoblasts. Figure 7A shows that CM of female, but not that of male, osteoclasts of cKO mutants increased BrdU incorporation compared to that of CM of osteoclasts of WT littermates of corresponding sex. However, neither CM of osteoclasts of male cKO mutants nor that of female cKO mutants significantly affected cellular ALP activity.

Information of cell proliferation and/or osteoblastic differentiation does not yield functional evidence for an effect on bone formation. Thus, we also compared the ability of the CM to promote mineralized nodule formation in cultured osteoblasts. Figure 7B shows that CM of osteoclasts of male cKO mutants formed smaller mineralized nodules than CM of osteoclasts of male WT littermates. Conversely, CM of female cKO osteoclasts formed larger mineralized nodules than CM of osteoclasts of female WT littermates. These findings suggest that *Grem1*-deficient osteoclasts of female enhanced, while those of male mutant mice reduced, the release of soluble factors to promote mineralization of bone nodules.

## Discussion

This study clearly demonstrated that osteoclasts expressed gremlin-1 and its expression was greatly upregulated upon osteoclastic differentiation, but the functional role of gremlin-1 in osteoclasts remains undefined. We sought to evaluate a novel hypothesis that osteoclast-derived *Grem1* has regulatory functions in the skeletal response to calcium stress using an osteoclastic *Grem1* cKO mouse model, which was based in parts on our observations that calcium depletion upregulated *Grem1* expression in bone and repletion rapidly returned bone *Grem1* level back to and even below the basal level. Calcium repletion is known to rapidly induce osteoclast apoptosis [5, 28]. Thus, the swift and large decline in bone *Grem1* level upon calcium repletion was probably due to the rapid apoptosis of osteoclasts. These findings also indicate that mature osteoclasts is a major cellular source of bone gremlin-1 during calcium depletion.

Conditional disruption of *Grem1* gene in *Ctsk*-expressing mature osteoclasts did not alter developmental growth and longitudinal bone growth. This is contrary to the impaired developmental growth and longitudinal bone growth seen in global *Grem1* null mutants [18]. Gremlin-1 is a potent antagonist of the BMP signaling, which is essential for epiphyseal growth plate development [29] and developmental bone growth [30]. The lack of an effect of deficient osteoclastic *Grem1* expression may imply that osteoclast-derived gremlin-1 may not be a key antagonist to the BMP-dependent endochondral bone formation during growth. However, *Ctsk*-expressing mature osteoclasts under basal conditions represent only a very small subpopulation of cells in the bone cavity. It is conceivable that the small number of mature osteoclasts might not have secreted enough gremlin-1 to be a key regulator of longitudinal bone growth. In addition, deletion of *Grem1* in *Ctsk*-expressing osteoclasts did not affect basal trabecular bone mass, density, or architecture, which differs from the transient increases in basal trabecular bone mass and density seen in mutant mice with conditional deletion of *Grem1* in osteocalcin-expressing osteoblasts [15]. While conditional deletion of osteoblastic *Grem1* promoted osteoblast differentiation and bone formation [15], disruption of *Grem1* in osteoclasts had no effects on basal BFR. Contrary to mature osteoclasts, osteoblasts represent a large cell population in the bone cavity. It is possible that the observed difference between the two *Grem1* cKO mouse strains was due to the difference in the relative amounts of gremlin-1 released by osteoblasts as opposed to that released by mature osteoclasts. Regardless of the reasons, this study indicates that osteoclast-derived gremlin-1 is not indispensable to basal bone formation.

This study disclosed several intriguing findings regarding the skeletal response to calcium depletion in these mice: first, female WT littermates in response to dietary calcium stress exhibited greater trabecular bone loss along with higher bone resorption than male WT littermates, indicating a sexual dimorphism in bone resorption response to calcium depletion. This observation is consistent with a previous report that showed a higher sensitivity to low calcium diet with greater osteoclast resorption and trabecular bone loss in female rats than male rats [24]. That female WT mice had greater bone resorption and smaller bone formation than male WT mice was confirmed by bone histomorphometry, indicating an opposite sexual disparity in bone resorption response to calcium depletion versus that in bone formation in WT mice.

The mechanistic reason for this complicated sexual dimorphism in skeletal response to calcium stress in WT mice is unclear. Sexual dimorphism has traditionally been attributed to the sex hormones. Thus, it is possible that sex disparity in the skeletal response to dietary calcium deficiency could also be due to the differential regulation of calcitropic hormones by sex hormones. Accordingly, estrogen increased calcium absorption in the intestines by stimulating renal 1α-hydroxylase activity [31], suppresses bone resorption by inducing osteoclast apoptosis [32], and promotes bone formation by increasing osteoblast survival [33]. Conversely, androgens increase urine calcium excretion [34] and increase bone formation through decreasing expression of the inhibitory IGFBP4 [35]. Thus, the opposite effects of estrogen versus testosterone on bone metabolism could in part contribute to the sexual dimorphism in skeletal response to calcium stress in WT mice. Moreover, the estrogen metabolism in female mice in situations of insufficient dietary calcium intake may cause a shift from one that involving the active 16α-hydroxyl pathways toward one that forms inactive estrogen metabolites [36–38], which would reduce the effective concentration of active estrogen. It is conceivable that calcium depletion in female WT littermates might have alleviated the inhibitory actions of estrogen and as such further enhanced the bone resorption. However, a recent study suggested that the greater sensitivity of the skeleton to calcium stress in female WT mice could be due to higher expression of genes associated with osteocytic osteolysis and osteoclastic resorption in osteocytes compared to male WT mice [39]. Hence, much additional work would be needed to better define the mechanism leading to sexual dimorphism in the skeletal response to calcium depletion in WT mice.

Second, the extent of trabecular bone loss to calcium depletion and osteoclastic resorption response were greater in male cKO mutant mice than in male WT littermates. This enhanced osteoclast resorption response to calcium stress in cKO mutants was sex-dependent and was restricted to male cKO mutants. Accordingly, unlike female WT littermates, female cKO mutants not only did not show an enhanced sensitivity but exhibited a reduced skeletal sensitivity to the calcium stress, in that calcium depletion caused substantially less trabecular bone loss, yielded smaller increase in OC.Pm/B.Pm, and failed to elicit an increase in NOC/B.Pm in female cKO mutants. Accordingly, there was a paradoxical, opposite sexual disparity in the skeletal response to calcium stress in male versus female cKO mutants.

Understanding the molecular mechanism contributing to this intriguing sex-dependent contrasting skeletal phenotype to calcium stress is essential as it could have important implications in our understanding of the regulatory role of osteoclastic-derived gremlin-1 in bone homeostasis. Because the contrasting sex-dependent skeletal response to calcium deficiency is seen in *Grem1* cKO mutant mice, and sex hormone is likely to play a role in sexual dimorphism in the skeletal response to calcium depletion in cKO mutants, we postulate that the molecular mechanism could in parts be related to the complicated interactions among gremlin-1, calcium and sex hormones (particularly estrogen) in the overall regulation of bone homeostasis. Accordingly, calcium depletion in mice promotes osteoclast differentiation, maturation, and activity [1–3], which then leads to rapid and large increases in the number of mature osteoclasts along the bone surface. Enhanced maturation and increased number of osteoclasts would increase their expression and local secretion of gremlin-1, which would then suppress the BMP signaling that stimulates osteoclastic resorption [40]. Thus, the gremlin-1-mediated suppression of the BMP signaling would reduce bone resorption in response to calcium depletion. In male cKO mutants without the influence of estrogen, deficient expression of *Grem1* would minimize or even eliminate the gremlin-1-mediated suppression of osteoclastic resorption and would thereby further enhance osteoclastic resorption and trabecular bone loss. In female WT mice, estrogen can downregulate *Grem1* expression through an estrogen receptor-dependent mechanism [41], which leads to upregulation of the BMP signaling-mediated osteoclast differentiation and bone resorption [40] and the observed further increase in bone resorption in response to calcium depletion. In addition, it has been reported that estrogen-related receptor α (ERRα) in breast cancer cells interacted with the promoter of *Grem1* to promote expression of *Grem1*, which in turn enhanced the ERRα promoter activity through phosphorylation and activation of EGFR (an upstream regulator of ERRα) to form a positive feedback loop [42]. The ERRα signaling has been shown to promote osteoclast differentiation and bone resorption [43], and deficient expression of ERRα in female mice rendered them resistant to age or estrogen deficiency-induced bone loss [44]. Thus, we postulate that deficient expression of *Grem1* in female cKO mice may reduce ERRα expression in their osteoclasts, which would lead to their relative unresponsiveness to calcium depletion with respect to an increased osteoclast resorption and trabecular bone loss. We should emphasize that our proposed mechanism did not consider testosterone, because there is currently not much information in the literature about interactions between gremlin-1 and the testosterone levels, metabolism, or functions. Nevertheless, we cannot rule out the possibility that testosterone may also have a contributing role.

Interaction between gremlin-1 and sex steroids might not be the only mechanism contributing to the complicated sex-dependent sexual disparity in the skeletal response to calcium depletion. For example, recent investigations have implicated important contributions for sex chromosomes to sexual dimorphism in osteoporosis by regulating the functions and life span of the various bone cells in regulating musculoskeletal mass and bone strength phenotype [45]. It has also been demonstrated that sex hormones and sex chromosomes each make independent contribution to the sex-dependent disparity in bone mass and strength [46]. Thus, it is likely that interactions between gremlin-1 and genes on the male and female sex chromosome, respectively, could also have important contributions. There is also evidence that bone marrow multipotent stromal cells exhibited intrinsic sex-linked variations in their osteogenic differentiation potential [47]. Other non-sex hormones-related factors may contribute to the molecular basis for the contrasting sex-dependent differences in the skeletal response to calcium deficiency in *Grem1* cKO mice.

Lastly, these cKO mutant mice also displayed sex disparity in their bone formation response to calcium depletion. Accordingly, male cKO mutants had much smaller BFR than male WT littermates in response to calcium depletion, whereas female cKO mutants displayed greater BFR than female WT littermates. It has been documented that osteoclasts released soluble osteoanabolic factors to mediate the coupled bone formation process [8–10]. In this regard, the relative bone nodule formation-stimulating activity in CM of osteoclasts of male cKO mutants was less than that of CM of osteoclasts of male WT littermates. Conversely, the increase in bone nodule formation activity in CM of osteoclasts of female cKO mutants was much greater than that of osteoclasts of female WT littermates. The sex-dependent difference in soluble osteoanabolic activity in CM of osteoclasts of male and female cKO mutants were consistent with the observed sex disparity in their bone formation response to calcium depletion. We interpret these findings as to that *Grem1* expression in osteoclasts of mutant mice may regulate the relative amounts of the release of one or more of such soluble osteoanabolic factors from osteoclasts in a sex-specific manner, and thereby indirectly affects the coupled bone formation in these cKO mutant mice. Gremlin-1 also regulates osteoblastic differentiation through the BMP signaling and proliferation via the canonical Wnt signaling. Thus, the possibility that gremlin-1 released by mature osteoclasts may act directly on osteoblasts to regulate the coupled bone formation cannot be ruled out.

A key limitation of this study is the use of *Ctsk*-Cre transgenic mice to generate *Grem1* cKO mutant mice. While *Ctsk* is a widely-used osteoclast-specific promoter to guide Cre recombinase activity for gene deletion in mature osteoclasts [48], *Ctsk* is also expressed in other skeletal cells at various levels, such as mature osteoblasts [49], osteocytes [49–51], skeletal stem cells [49, 52], and tendon cells [53] in addition to mature osteoclasts. Thus, although we interpret that the observed effects were due largely to the deficient expression of *Grem1* in *Ctsk*-expressing mature osteoclasts, we cannot ignore the possibility that some of the observed effects may also be off-target effects due to the deletion of *Grem1* in other skeletal cells, particularly osteoblasts and osteocytes. However, conditional deletion of *Grem1* in osteocalcin-expressing osteoblasts yielded different skeletal response [15] than *Grem1* deletion in *Ctsk*-expressing cells. It is unlikely that the observed skeletal effects were caused by off-target effects of the unintended deletion of *Grem1* in osteoblasts. On the other hand, calcium stress, such as calcium depletion and lactation, could trigger substantial amounts of perilacunar/canalicular resorption and cortical bone loss and that osteocytes play a major role in the osteocytic perilacunar/canalicular resorption [50, 54]. Mature osteoblasts expressed substantial amounts of *Grem1* [15] and thereby osteocytes could also express *Grem1*. Because calcium stress, such as lactation and calcium depletion, significantly upregulated *Ctsk* expression in osteocytes [55], it is possible that calcium depletion could have increased the *Ctsk*-mediated deletion of osteocytic *Grem1* in the mutant mice. Thus, we have to consider the possibility that the unintended deletion of osteocytic *Grem1* could contribute to at least some of the observed effects. The fact that the sex disparity in the response to calcium depletion was seen in trabecular but not cortical bone may support the possibility that the unintended deletion of *Grem1* in osteocytes could somehow be responsible for the lack of sex-dependent effects in cortical bone. Further studies are needed to evaluate this possibility.

Another key limitation is that the study focused on the effects of gremlin-1 on endochondral bone. Accordingly, we will in future investigate whether conditional deletion of *Grem1* in mature osteoclasts would also yield similar contrasting sex-dependent phenotype in intramembranous bone in male and female *Grem1* cKO mutant mice, since we cannot simply assume that gremlin-1 would act similarly in intramembranous bone as in endochondral bone during calcium depletion.

In summary, this study demonstrates for the first time that osteoclast-derived gremlin-1 may have an important regulatory role in the skeletal response to dietary calcium depletion. The nature of the regulatory role of osteoclastic gremlin-1 in the skeletal response to calcium depletion is highly complex and may involve complicated interactions among gremlin-1, estrogen, and calcium. These putative intricate interactions are probably major contributing factors to conferring the converse sex-dependent differences in osteoclastic and bone loss responses to calcium stress in osteoclastic *Grem1* cKO mutant mice compared to the age- and sex-matched WT littermates. The physiological significance of these findings is not yet clear. It has been well established that the osteoclastic release of osteoanabolic factors have important mediating role in the bone coupling process [8–10]. This study presents strong, albeit circumstantial, evidence that gremlin-1 could be a potent osteoclast-derived soluble osteoanabolic factor. If confirmed, osteoclast-derived gremlin-1 could be a novel sex-dependent osteoclast-derived “bone coupling” factor that is released by active osteoclasts to act directly on nearby osteoblasts at or near the end of the resorption phase of the bone remodeling process to in part mediate the couple bone formation. Calcium deficiency is a common pathology of postmenopausal [56] and senile osteoporosis [57]. It follows that osteoclast-derived gremlin-1 could be a novel therapeutic target for the development of novel treatment for postmenopausal and senile osteoporosis.

## Supplementary Information

Below is the link to the electronic supplementary material.

Supplementary file1 (DOCX 1918 KB)

## Data Availability

The research data that support the findings of this study is stored at an approved storage facility within the Loma Linda VA Healthcare system and are available from other investigators for review upon reasonable request and approval by the Loma Linda VA Healthcare system.

## Declarations

### Disclaimers

The views, opinions and/or findings contained in this report are those of the authors and should not be construed as an official position, policy or decision of the US Army or the United States government, unless so designated by other documentation. The funders had no role in study design, data collection and analysis, decision to publish, or preparation of the manuscript.
