# Supplementary material for: Conditional Deletion of Gremlin-1 in Cathepsin K-expressing Mature Osteoclasts Altered the Skeletal Response to Calcium Depletion in Sex-Dependent Manner
Source: Calcif Tissue Int. 2025 Jan 9;116(1):28. doi: 10.1007/s00223-024-01337-7 (PMC11717885; doi:10.1007/s00223-024-01337-7)
Supplement: Supplementary file 1 — Supplementary file1 (DOCX 1918 KB) [file 223_2024_1337_MOESM1_ESM.docx]

**Supplemental Information**

**to**

**Conditional Deletion of Gremlin-1 in Cathepsin K-expressing Mature Osteoclasts Altered the Skeletal Response to Calcium Depletion in Sex-Dependent Manner**

Matilda H.-C. Sheng.^1,2^ Charles H. Rundle,^1,2^ David J. Baylink,^2^ and Kin-Hing William Lau,^1,2^

^1^ Jerry L. Pettis Memorial VA Medical Center, VA Loma Linda Healthcare System, Loma Linda, California, U.S.A.; and

^2^ Department of Medicine and Biochemistry, Loma Linda University School of Medicine, Loma Linda, California, U.S.A.





**Supplemental Figure S1. Schematic illustration of the breeding strategy for generating the conditional *Grem1* knockout mouse strain in *Ctsk*-expressing mature osteoclasts.**


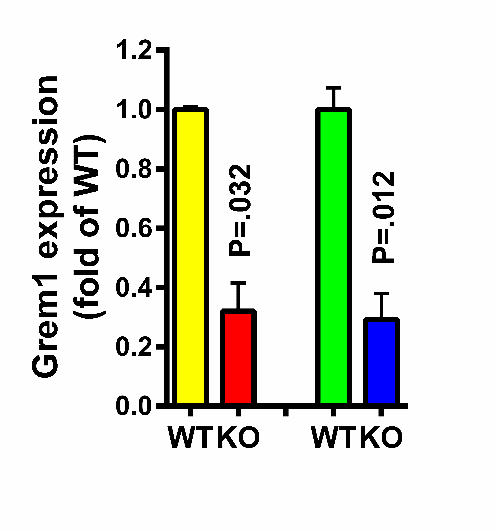

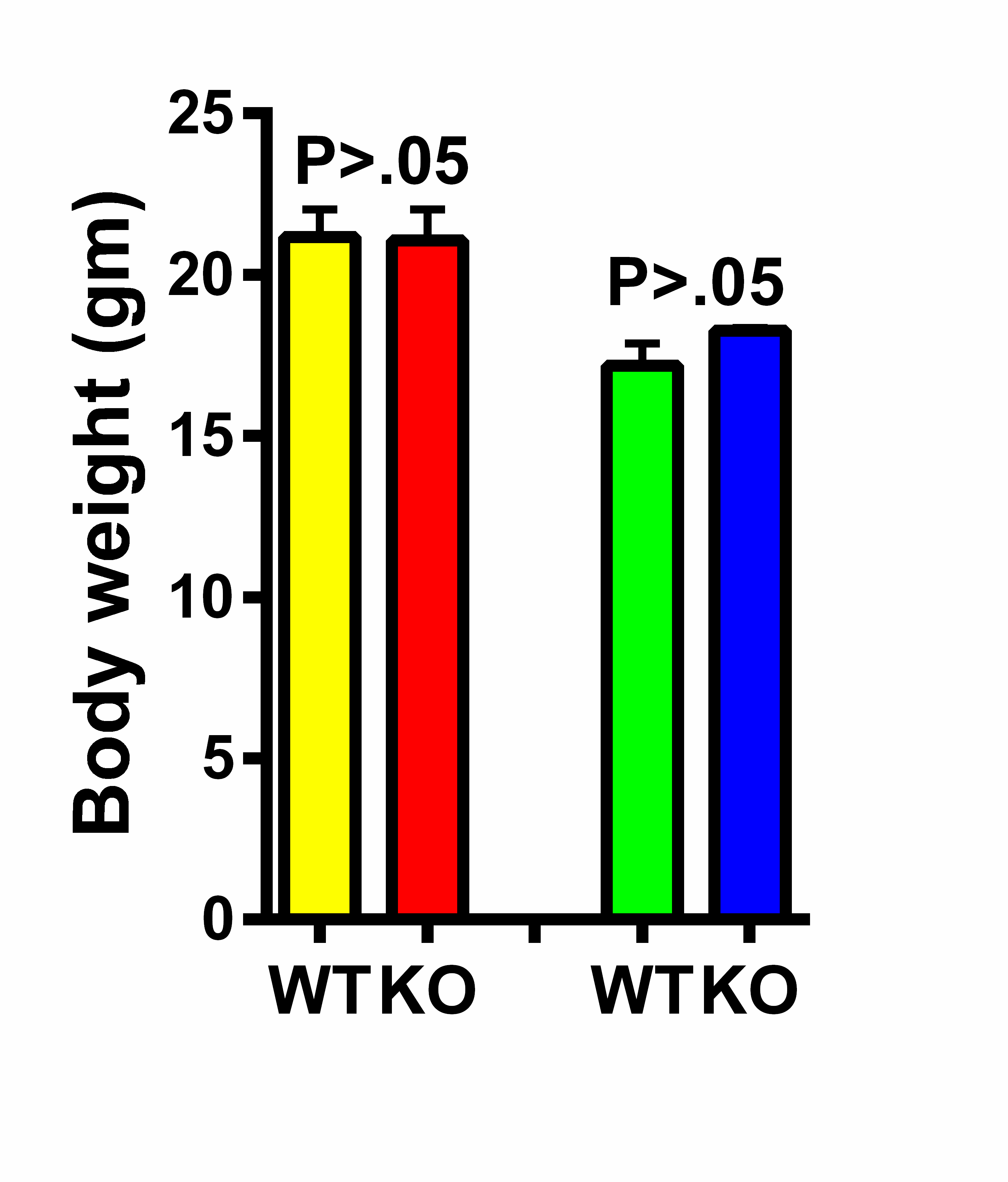

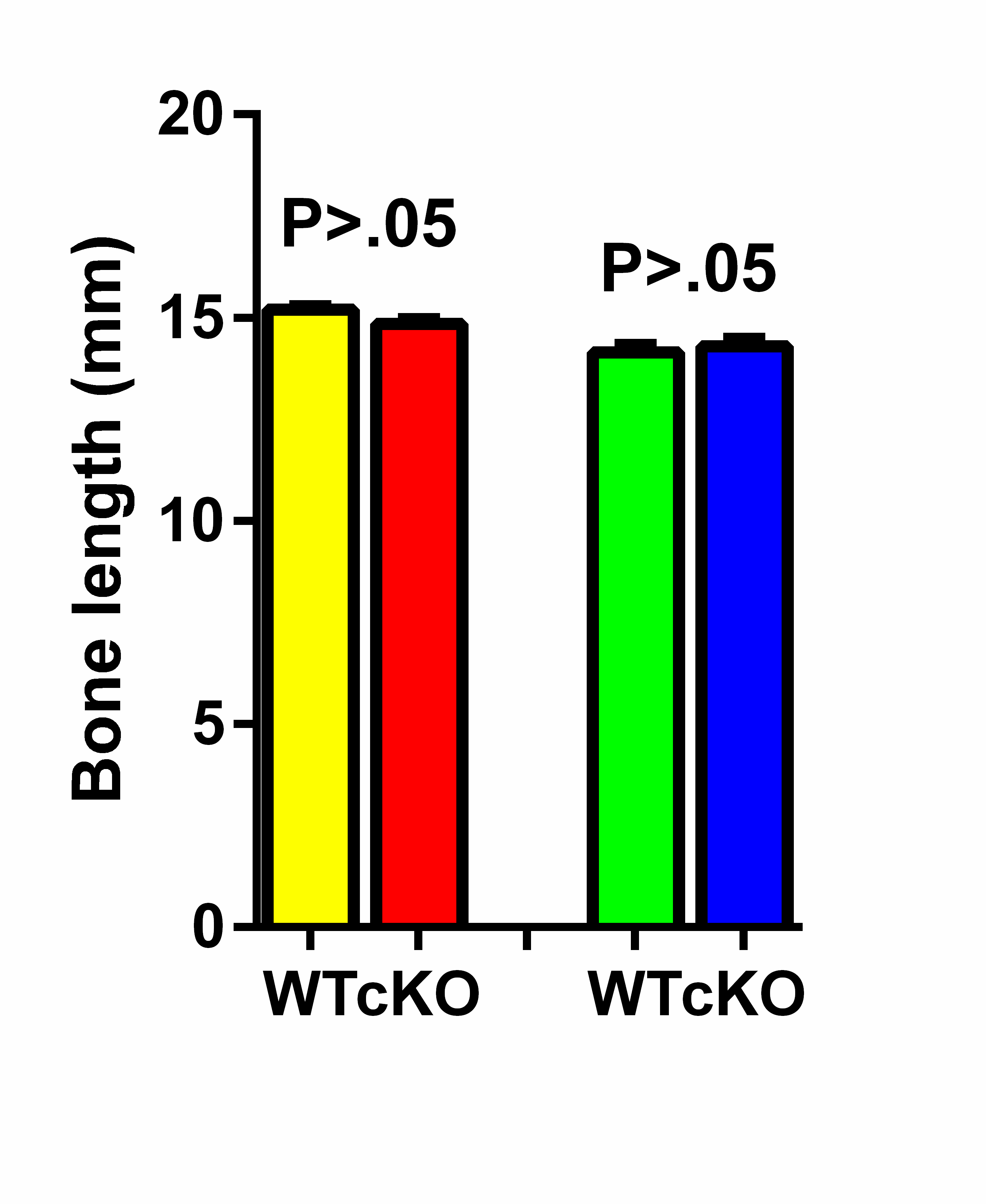


**B**

**C**

**A**

**Males**

**Females**

**Males**

**Females**

**Males**

**Females**

**Supplemental Figure S2. Mutant mice with conditional deletion of *Grem1* in *Ctsk*-expressing cells expressed greatly reduced levels of *Grem1* mRNA (A) in bone extracts of male and female *Grem1* cKO mutants as well as male and female WT littermates, and it had no effects on body weight (B) or femur length (C).** A: *Grem1* mRNA levels normalized by β-actin were determined by RT-qPCR of bone extracts of 4 male and 4 female *Grem1* cKO mutants as well as 4 male and 4 female WT littermates. B: Body weight was determined with an electronic balance in 9 male and 5 female *Grem1* cKO mutants as well as 8 male and 5 female WT littermates, and C: length of femurs was determined with a digital caliper in 6 male and 7 female *Grem1* cKO mutants as well as 7 male and 8 female WT littermates. Results are shown as mean ± SEM. Statistical significance was determined with two-tailed Student’s t-test. P>0.05 = not significant.


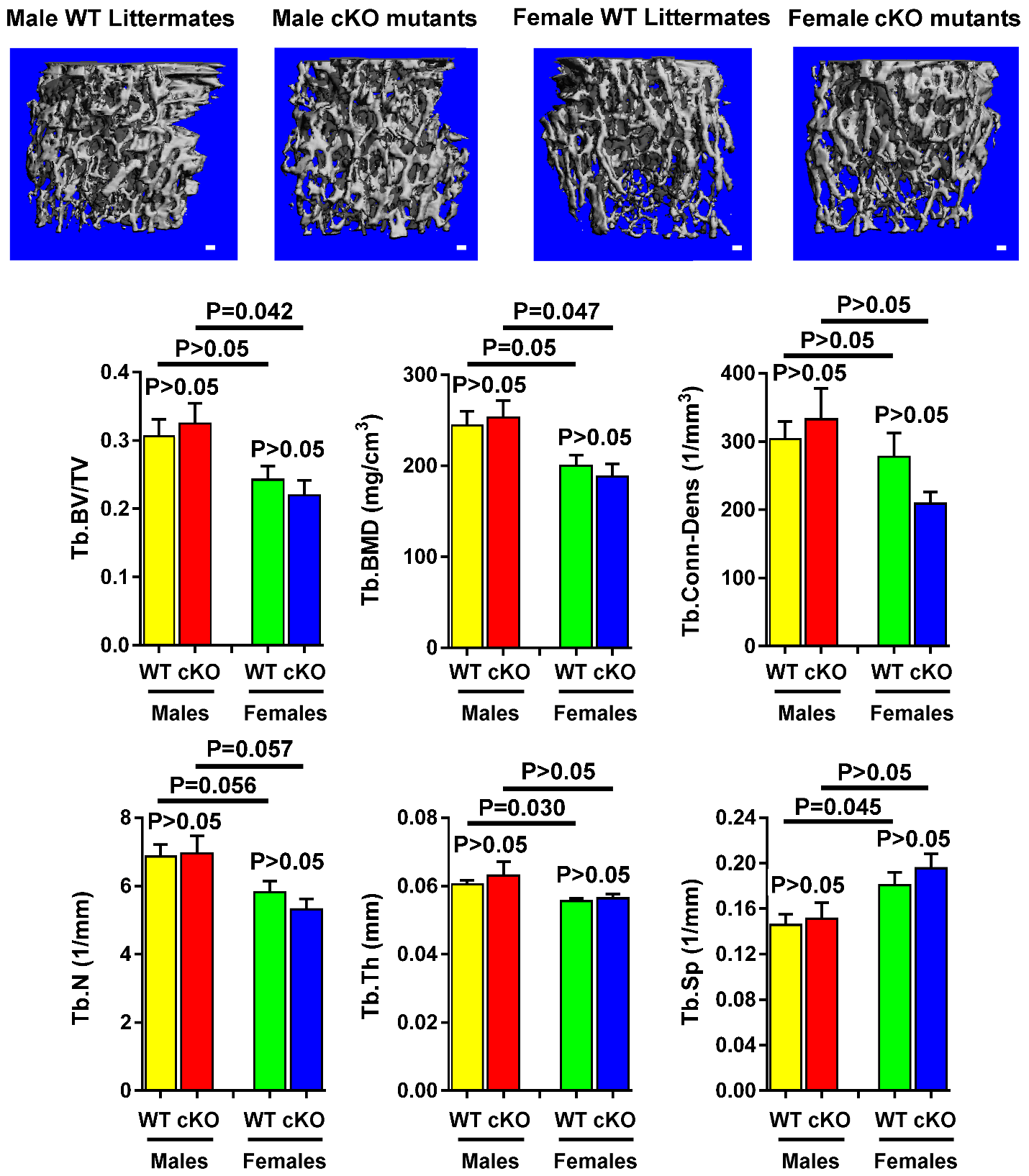


**Supplemental Figure S3. Effects of conditional deletion of *Grem1* in *Ctsk*-expressing cells on trabecular bone parameters in male and female mutant mice under basal conditions at 6 weeks of age.** Trabecular bone parameters were measured by µ-CT at secondary spongiosa of the distal femur as described in Methods. Top: Photomicrograph of three-dimensional reconstruction of trabecular bone mass at the AP view of a representative mouse of each group. Bottom: Summary of µ-CT trabecular bone parameters. Results are shown as mean ± SEM, n=6-7. Statistical analyses were performed with two-tailed Student’s t test. P>0.05 = statistically not significant.


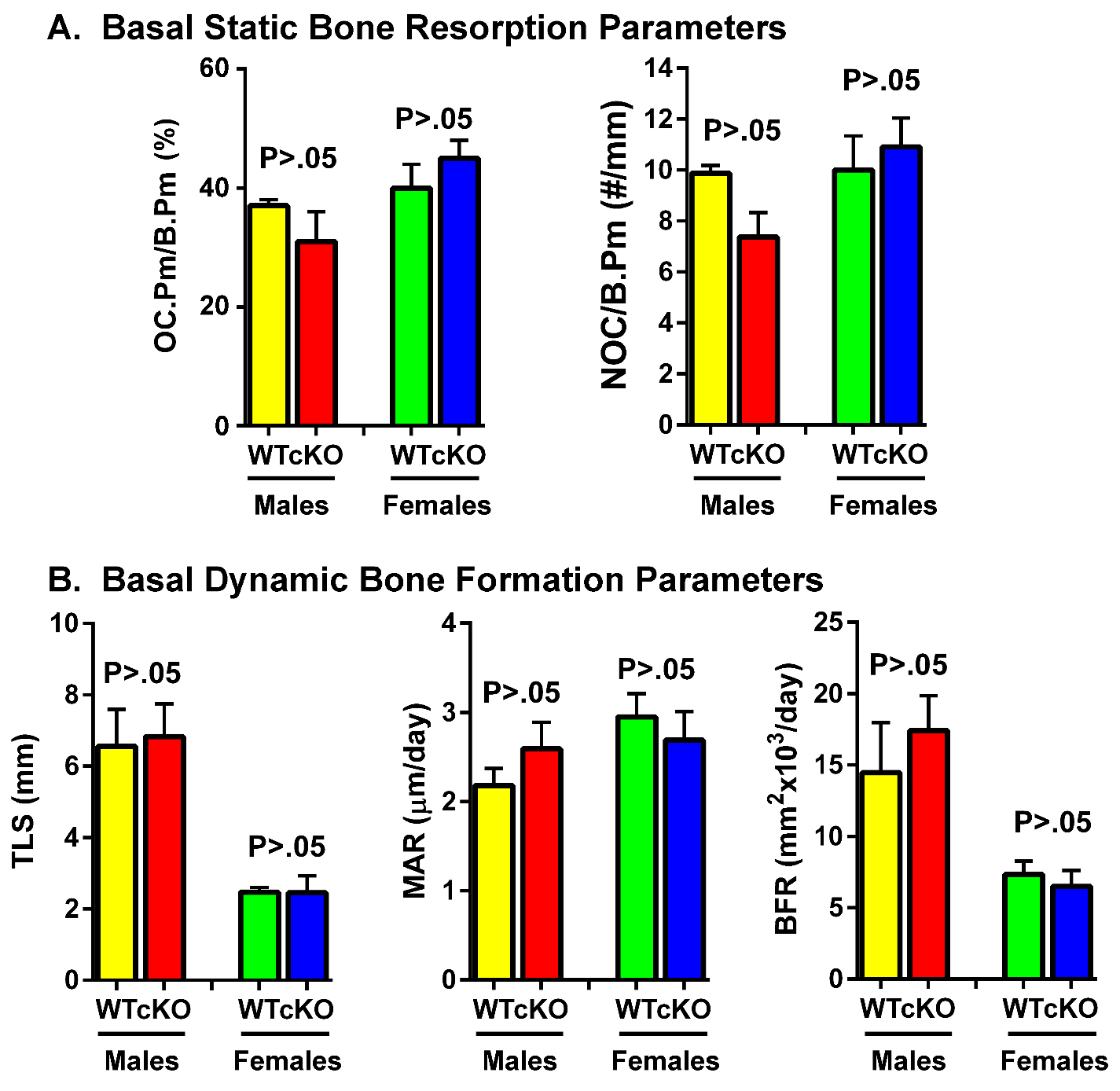
**Supplemental Figure S4. Effects of conditional deletion of *Grem1* in *Ctsk*-expressing osteoclastic cells on basal static bone resorption and dynamic bone formation parameters in male and female *Grem1* cKO mutant mice with those in WT littermates of corresponding sex at 6 weeks of age.** Static bone resorption and dynamic bone formation parameters were determined by bone histomorphometry as described in Methods with the exception that femurs were embedded in paraffin instead of Glycol methacrylate. Results are reported as mean ± SEM (n=5-6 per group). Statistical analyses in both panels were performed with two-tailed Student’s t test. P>0.05 = statistically not significant.
